# Influence of Hamstring Muscles Extensibility on Spinal Curvatures and Pelvic Tilt in Highly Trained Cyclists

**DOI:** 10.2478/v10078-011-0035-8

**Published:** 2011-10-04

**Authors:** José M. Muyor, Fernando Alacid, Pedro A. López-Miñarro

**Affiliations:** 1 Department of Physical Education. University of Almería. Spain.; 2 Department of Physical Activity and Sports. University of Murcia. Spain.; 3 Department of Physical Education. University of Murcia. Spain.

**Keywords:** straight leg raise, sit-and-reach, posture, spine, cycling

## Abstract

The purpose of this study was to determine the influence of hamstring muscles extensibility in standing, maximal trunk flexion with knees extended and on the bicycle in lower handlebar-hands position of highly trained cyclists. Ninety-six cyclists were recruited for the study (mean ± SD, age: 30.36 ± 5.98 years). Sagittal spinal curvatures and pelvic tilt were measured in the standing position, maximal trunk flexion with knees extended (sit-and-reach test) and while sitting on a bicycle in lower handlebar-hand position using a Spinal Mouse system. Hamstring muscles extensibility was determined in both legs by passive straight leg raise test (PSLR). The sample was divided into three groups according to PSLR angle: (1) reduced extensibility (PSLR < 80º; n = 30), (2) moderate hamstring extensibility group (PSLR = 80º – 90º; n = 35), and (3) high hamstring extensibility (PSLR = > 90º; n = 31). ANOVA analysis showed significant differences among groups for thoracic (p < 0.001) and pelvic tilt (p < 0.001) angles in the sit-and-reach test. No differences were found between groups for standing and on the bicycle position. Post hoc analysis showed significant differences in all pairwise comparisons for thoracic angle (p < 0.01) and pelvic angle (p < 0.001) in the sit-and-reach test. No differences were found in lumbar angle in any posture. In conclusion, the hamstring muscles extensibility influence the thoracic and pelvic postures when maximal trunk flexion with knees extended is performed, but not when cyclists are seated on their bicycles

## Introduction

Three muscles (*semimembranosus*, *semitendinosus* and *biceps femoris)* comprise the hamstring muscle group. This muscle group is attached to the ischial tuberosity of the pelvis. The pelvis is considered the base of the spine and its anteroposterior orientation affects the spinal sagittal curvatures (Levine and Whittle, 1996). Increased hamstring tension influences pelvic posture (Congdon et al., 2005; Dewberry et al., 2003) and spinal curvatures (López-Miñarro and Alacid, 2010). Alterations in sagittal spinal curvatures increase intervertebral stress (Beach et al., 2005) as well as thoracic and lumbar intradiscal pressure (Nachemson, 1976; Polga et al., 2004; Sato et al., 1999; Wilke et al., 1999), predisposing the subjects to spinal disorders (McGill, 2002).

Several studies have analyzed the interrelation between passive hamstring extensibility and spinal curvatures. Passive extensibility of skeletal muscles is defined as the ability of skeletal muscles to lengthen without muscle activation (Gajdosik, 2001). No association has been described between hamstring extensibility with spinal and pelvic posture while standing (Gajdosik et al., 1994; López-Miñarro and Alacid, 2010). However, most studies have found a relationship between hamstring flexibility and trunk posture when trunk flexion movements are performed. Li et al. (1996) found that reduced hamstring muscles extensibility was associated with greater relative lumbar flexion during forward bending. Gajdosik et al. (1994) reported that short hamstrings were associated with decreased pelvic flexion and lumbar angle and increased thoracic flexion in the toe-touch test. Carregaro and Gil (2009) reported increased trunk angles in subjects with reduced hamstring flexibility during manual handling tasks. López-Miñarro and Alacid (2010) found that hamstring muscles extensibility influences the thoracic and pelvic postures of young paddlers in the sit-and-reach test. However, they did not evaluate the specific position of athletes during their training.

Cycling is characterised by a sitting position with the trunk flexed to reach the handlebars of the bicycle. The knee is slightly flexed when the pedal is situated in the lower position (around 20 degrees). The posture maintained on the bicycle may influence spinal curvatures. Rajabi et al. (2000) found greater standing thoracic kyphosis in cyclists than in sedentary individuals. Usabiaga et al. (1997) showed a change from lumbar lordosis to lumbar kyphosis when the cyclists were seated on their bicycle. Muyor et al. (2011) found greater lumbar flexion in cyclists when handlebar-hands position is situated farthest and lowest with respect to the saddle of the bicycle. However, these studies have not analyzed the influence of hamstrings extensibility on spinal curvatures and pelvic position. McEvoy et al. (2007) found greater anterior pelvic tilt in comparison with sedentary subjects when sitting on the floor with extended knees. This difference could be related to greater hamstrings extensibility in cyclists.

Because no studies have analyzed the relation between hamstring muscles extensibility and pelvic and spinal posture in cyclists, the objective of this study was to determine the influence of hamstrings muscles extensibility of highly trained cyclists on spinal curvatures and pelvic position in various arrangements of the body.

## Material and methods

Ninety-six cyclists were recruited for the study (mean ± standard deviations, age: 30.36 ± 5.98 years; height: 1.76 ± 0.06 m; body mass: 76.05 ± 9.28 kg). The inclusion criteria were 1) daily training on the bicycle between two and four hours; 2) training between three and six days per week; and 3) at least five years of training experience. The exclusion criteria were 1) a history of spinal or hamstrings pain in the three months prior to the study; 2) a history of spinal or hamstrings surgery; or 3) a medically diagnosed spinal disorder. All participants were instructed to avoid strenuous training and physical activity 24 hours prior to the study.

### Procedures

**Figure 1:**
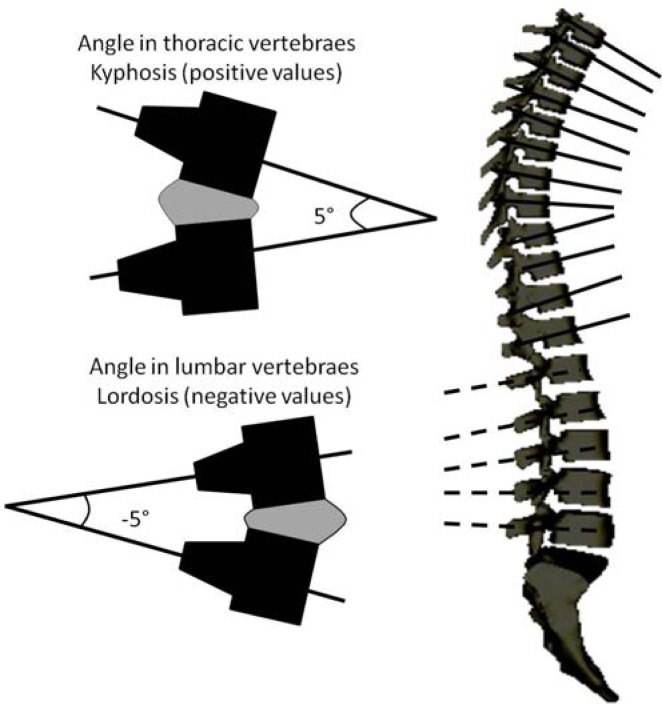
Schematic of angles calculated by Spinal Mouse

An Institutional Ethical Committee approved the study and all participants were informed of the procedures and signed an informed consent prior to the measurements. Hamstring muscles extensibility was determined in both legs using the passive straight leg raise test. This test has been provided as a clinically valid indication of hamstring muscle length (Gajdosik et al., 1990; Gajdosik, 1991a,b). Sagittal spinal curvatures and pelvic tilt were measured in the standing position, maximal trunk flexion with knees extended (sit-and-reach test) and sitting on a bicycle in lower handlebar-hands position using a Spinal Mouse^®^ system (Idiag, Fehraltdorf, Switzerland). The Spinal Mouse was guided along the midline of the spine. Two rolling wheels follow the contour of the spine, and distance the angle measures are communicated from the device to a base station positioned approximately 1–2m away and interfaced to a personal computer. Data is sampled every 1.3 mm as the mouse is rolled along the spine, giving a sampling frequency of approximately 150 Hz. The average total length of the spine is 550 mm and the time required for measurements of the whole length is 2–4 s; thus, approximately 423 measurements are made over 3 s. This information is then used to calculate the relative positions of the sacrum and vertebral bodies of the underlying bony spinal column using an intelligent, recursive algorithm (Mannion et al., 2004) (Figure 1).

The measurements were made in randomized order in a single session. No warm-up or stretching exercises were performed by the subjects prior to the test measurements. The subjects were allowed to rest briefly standing up for 5 minutes between measures. Each subject was evaluated wearing underwear and barefoot. The laboratory temperature was standardised at 24ºC.

Prior to taking measurements, the main researcher determined the position/location spinous process of C7 (starting point) and the top of the anal crease (end point) by palpation and marked the skin surface with a pencil. The Spinal Mouse was guided along the midline of the spine (or slightly paravertebrally in particularly thin individuals with prominent processus spinous) starting at the processus spinous of C7 and finishing at the top of the anal crease (approximately S3). For each testing position, the angle of the thoracic (T1-2 to T11-12) and lumbar (T12-L1 to the sacrum) spine and the inclination of the pelvis (difference between the sacral angle and the vertical) were recorded. In the lumbar curve negative values corresponded to lumbar lordosis (posterior concavity). With respect to the pelvic position, a value of 0º represented the vertical position. Thus, a greater angle reflected an anterior pelvic tilt and a lower angle (negative values) reflected a posterior pelvic tilt. Each measurement was repeated twice within a 20-sec trial. The average of the two trials was used.

### Measures

#### 

##### Hamstring muscles extensibility

**Figure 2:**
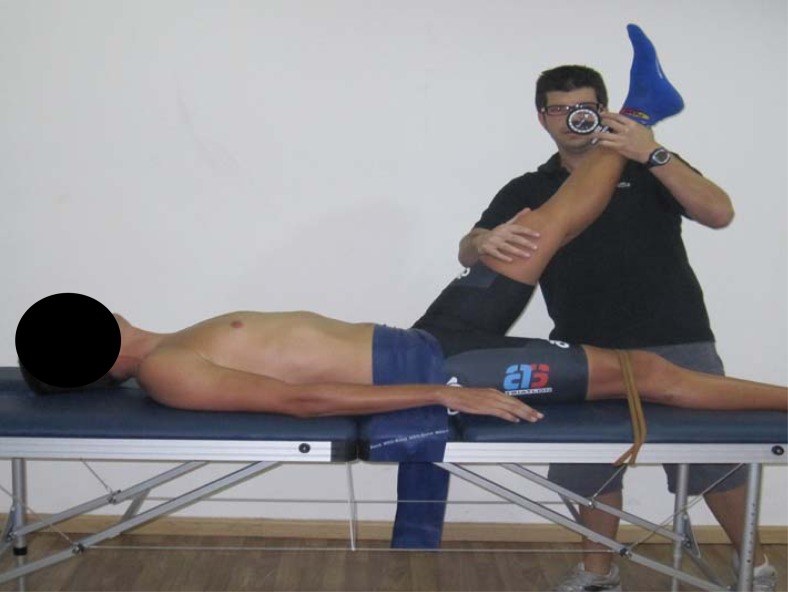
Passive straight leg raise (PSLR)

The criterion measure of hamstrings extensibility was determined by performing a passive straight leg raise (PSLR) on each limb in counterbalanced order. This test was made following the protocol described by López-Miñarro et al. (2010). Subjects were positioned in supine with the lower extremity in 0º of hip flexion, maintained by a Velcro strap secured to the table. While the participant was in the supine position a Uni-Level inclinometer (ISOMED, Inc., Portland, OR) was placed over the distal tibia. The participant’s leg was lifted passively by the tester into a hip flexion. The knee remained straight during the leg raise. Moreover, the pelvis was fixed to avoid the posterior pelvic tilt. Another Velcro strap was positioned over the anterior superior iliac spine. The criterion score of hamstring extensibility was the maximum angle (degree) read from the inclinometer at the point of maximum hip flexion (Figure 2). The ankle of the tested leg was restrained in plantar flexion to avoid adverse neutral tension (Gajdosik et al., 1985). The endpoint for the straight leg raise was determined by one or both of two criteria: a) the subjects reported pain in their hamstring muscles, and/or b) palpable onset of pelvic rotation. Two trials were given for each leg and the average of the two trials on each side was used for subsequent analysis.

#### Standing

The subject assumed a relaxed position, with the head looking forward, the arms hanging by the side, the knees normally extended and the feet shoulder-width apart.

#### Maximal trunk flexion with extended knees (sit-and-reach test)

Subjects were required to sit with knees straight and legs together so that the soles of the feet were flat against the end of a constructed sit-and-reach box (height = 32 cm). With palms down, subjects placed one hand on top of the other and slowly reached forward as far as possible. Subjects slid their hands along the box, with their knees kept as straight as possible, and held the resulting position for approximately five seconds while the spinal curvatures and pelvic inclination were measured.

#### Sitting on the bicycle

**Figure 3:**
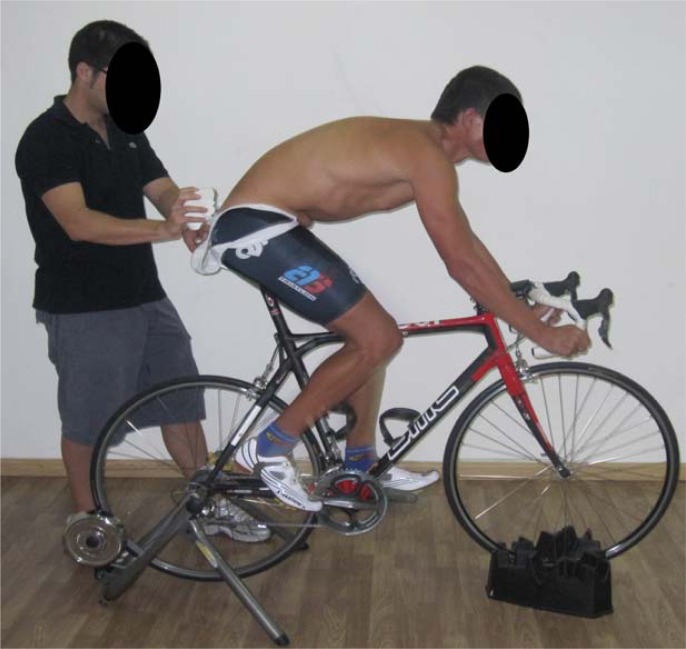
Sitting on the bicycle with lower handlebar-hands position

Subjects were asked to pedal in lower handlebar-hands position (Figure 3) for 5 min at a cadence of 95 rpm. Cyclists used their own road bicycles on an indoor wind trainer. Cyclists maintained their individual set-up in the bicycle.

### Statistical analysis

Intra-tester reliability of thoracic and lumbar curvatures and pelvic tilt was calculated in a previous pilot study. Twenty subjects who did not participated in the final sample were measured three times by the same tester in standing position on the floor, sitting on a stool, and prone lying in a single session. Intra-class correlation coefficients (ICC) with 95% confidence intervals (CI) were calculated. An ICCs upper or equal to 0.98 (95% CI: 0.98 – 0.99) were obtained for thoracic kyphosis, lumbar lordosis and pelvic tilt in all postures evaluated.

The left and right PSLR measurements were averaged. The sample was divided into three groups: reduced hamstrings extensibility group (Group A, PSLR < 80º; n = 30), moderate hamstrings extensibility group (Group B, PSLR = quitar espacios 80º – 90º; n = 35), and high hamstrings extensibility (Group C, PSLR = > 90º; n = 31).

Means and standard deviations (± SD) were quitar espacios calculated for all variables. The hypotheses of normality was analysed using the Kolmogorov-Smirnov test. One-way analysis of variance (ANOVA) was conducted to examine differences between groups for all dependent variables. Significant F-ratios were followed by Tukey’s post hoc analyses to examine pairwise group differences. The data were analyzed using the SPSS v.15.0. The level of significance was set at *p* ≤ 0.05.

## Results

The mean (± SD) PSLR values were 71.85 ± 5.89º, 84.71 ± 3.36º, and 99.98 ± 6.02º for groups A, B and C, respectively. The one-way ANOVA revealed significant differences between groups (*F* = 226.71, *p* < 0.001). *Post hoc* analysis showed significant differences in all pairwise comparisons (*p* < 0.001).

The descriptive data and ANOVA results for the dependent variables in the standing, sit-and-reach test, and sitting on the bicycle in lower handlebar-hands posture are presented in Table 1. *Post hoc* analysis showed significant differences in all pairwise comparisons for thoracic angle (*p* < 0.01) and pelvic inclination (*p* < 0.001) in the sit-and-reach test. No differences were found in lumbar angle for any posture. No significant differences between groups were detected in standing and on the bicycle (Table 1).

## Discussion

The purpose of this study was to determine the sagittal spinal curvatures and pelvic posture of cyclists according to their hamstring muscles extensibility. The main finding was that hamstring muscles extensibility influences the thoracic angle and pelvic position in maximal trunk flexion with knees extended. Cyclists with reduced hamstring extensibility showed greater thoracic angles and more posterior pelvic tilt in the sit-and-reach test. However, hamstring extensibility did not influence the spinal curvatures neither pelvic inclination in standing and sitting on the bicycle. These findings are in agreement with previous research reporting a relationship between hamstring extensibility and pelvic position and spinal postures in trunk flexion movements (Carregaro and Gil, 2009; Gajdosik et al., 1992; 1994; Li et al., 1996; Bellew et al., 2010). However, these studies evaluated non athlete populations.

Gajdosik et al. (1992; 1994) found that lower hamstring extensibility significantly reduces pelvic flexion in the toe-touch test whereas the thoracic curve was increased. In the current study similar results were found using the sit-and-reach test. Miñarro et al. (2007) found that toe-touch test generates a lower thoracic angle in comparison with the sit-and-reach test when the same subjects are evaluated with both tests. Because the hamstring muscles have their anatomical attachment on the ischial tuberosity, when maximal trunk flexion with knees extended is performed the pelvis rotates forward until the passive tension in the hamstrings increases significantly. At this point the hamstrings limit anterior pelvis rotation (Shin et al., 2004; Congdon et al., 2005). Recently, Bellew et al. (2010) reported that hamstring extensibility strongly correlated to pelvic rotation during maximal trunk flexion with knees extended. However, Norris and Matthews (2006) did not find any association between hamstring muscles extensibility and pelvic tilt in a student population, although they analyzed a submaximal flexion movement. In the current study no significant differences were found in spinal curvatures and pelvic tilt on the bicycle. Asknees are flexed to around 20º (de Vey Mestdagh, 1998) the hamstring extensibility did not influence the pelvic posture and spinal curvatures while sitting on the bicycle. Usabiaga et al. (1997) reported a change of lumbar lordosis to lumbar kyphosis when the cyclists were seated on their bicycles. A similar relation is reported here. Greater lumbar flexion was found in the high extensibility group, but no significant differences were detected between groups. Recently, Muyor et al. (2011a,b) found greater lumbar flexion in cyclists and non-athletes when handlebar-hands position is situated farthest and lowest with respect to the saddle of the bicycle. McEvoy et al. (2007) found that elite cyclists had a significantly greater anterior pelvic tilt angle than non-cyclists when tested in sitting with knees extended, although they did not evaluate hamstring extensibility. They justified their finding as a specific adaptation to a training position that may contribute to a greater anterior pelvic tilt.

In this study, cyclists with reduced hamstrings extensibility showed greater thoracic flexion in the sit-and-reach test. These results may be related to restricted anterior pelvic tilt (Gajdosik et al., 1994). If the pelvis is positioned in posterior pelvic tilt the subjects increase the thoracic flexion to reach greater distances in the sit-and-reach (López-Miñarro and Alacid, 2010). Reduced hamstrings flexibility could play a role in spinal stress. Previous studies have found increases in spinal load (Keller et al., 2005), disc pressure (Nachemsom, 1976; Polga et al., 2004; Sato et al., 1999; Wilke et al., 1999), creep deformation in lumbar tissues (Caldwell and Peters, 2009; Solomonow et al., 2003) and low back pain (Harrison et al., 2005; Smith et al., 2008) when static or cyclic flexion postures are performed. In this sense, the low back pain has been reported like the most common overuse injury in cyclists (Asplund et al., 2005; Clarsen et al., Marsden and Schwellnus, 2010; Salai et al., 1999). With regard to the standing position, Briggs et al. (2007) found that increases in standing thoracic kyphosis were associated with significantly higher spinal loads and trunk muscle forces. In the current study no significant differences were found in standing between groups, although the mean values of thoracic kyphosis in the three groups correspond to hyperkyphotic posture (> 45º) (Mejia et al., 1996).

With respect to sample selection, only cyclists without current or chronic history of low back pain were recruited. A high percentage of cyclists usually report low back pain, perhaps due to prolonged seated positions on the bicycle (Salai et al., 1999) or to an incorrect saddle angle (Marsden and Schwellnus, 2010). Low-back pain was an exclusion criterion because previous studies have shown that low-back pain is related to changes in lumbopelvic rhythm (Esola et al., 1996) and induces modifications in the position on the bicycle (Burnett et al., 2004; De Vey Mestdagh, 1998; Salai et al., 1999). Future studies should investigate the relation between low-back pain, spinal curvatures and pelvic posture on the basis of hamstrings extensibility.

In conclusion, hamstring muscles extensibility influences the thoracic kyphosis and pelvic tilt when maximal trunk flexion with knees extended is performed. High hamstring extensibility was associated to lower thoracic angles and more anterior pelvic tilt. However, the hamstring muscles extensibility does not have any influence in standing and on the bicycle with the lower handlebar-hands position.

## Figures and Tables

**Table 1:** Descriptive statistics (Mean ± SD) of the dependent variables for reduced hamstrings extensibility (PSLR < 80º), moderate hamstrings extensibility (PSLR = 80º – 90º), and high hamstrings extensibility (PSLR > 90º) groups

	Passive straight leg raise			*F*	*P*
	Group A < 80º (n = 30)	Group B 80º – 90º (n = 35)	Group C > 90º (n = 31)		
Thoracic angle standing	46.86 ± 7.59º	48.97 ± 10.79º	45.71 ± 7.96º	1.12	0.331
Lumbar angle standing	−25.10 ± 5.28º	−27.34 ± 6.88º	−25.29 ± 6.94º	1.23	0.295
Pelvic tilt standing	11.07 ± 5.61º	12.51 ± 4.25º	11.90 ± 5.23º	0.67	0.513
Thoracic angle SR	64.73 ± 7.18º	61.86 ± 10.38º	52.97 ± 12.27º	11.16	0.000
Lumbar angle SR	29.80 ± 7.24º	30.40 ± 8.39º	33.10 ± 9.11º	1.38	0.255
Pelvic tilt SR	−19.18 ± 7.78º	−8.68 ± 9.86º	−2.13 ± 8.27º	32.22	0.000
Thoracic angle bicycle	41.03 ± 9.69º	38.80 ± 10.09º	39.65 ± 10.00º	0.41	0.664
Lumbar angle bicycle	25.37 ± 7.70º	24.14 ± 6.98 º	26.23 ± 6.75º	0.71	0.494
Pelvic tilt bicycle	33.53 ± 6.55º	35.40 ± 6.84º	35.71 ± 6.51º	0.96	0.382

Mean ± SD; SR: sit-and-reach. Negative values reflect lumbar concavity.
